# American Academy of Dental Science

**Published:** 1886-02

**Authors:** 


### AMERICAN ACADEMY OF DENTAL SCIENCE.

The regular monthly meeting of the Academy was held on Jan. 6th, at the residence of Dr. E. H. Smith, Dartmouth St., Boston. After the passage of resolutions of respect to the memory of the late Dr. Riggs, an associate member, the subject of the evening's discussion, "Crowning Teeth," was taken up. Dr. T. H. Chandler, who was to have opened the discussion, was absent. Dr. Merriam exhibited some wire, such as he uses in his own practice in crowning teeth, and stated that he has for some years set all of his crowns with gutta-percha. He thought there were some serious objections to the Richmond Crown, and one is that there are cases which need immediate attention and could not be delayed until a Richmond Crown could be set.

*Dr. Clapp*—Said that three years ago a patient came to him with a very large bicuspid tooth broken off. The pulp canal had been previously filled with gold, and was of large size. He inserted a crown, securing it with gutta-percha. In a short time the patient came back, and the root had split in twain. He would like to inquire whether there is sufficient expansion taking place in gutta-percha to crack open a tooth.*

*Dr. Wilson*—Had noticed a tendency in all dead teeth to crack open, and it was, he thought, due to a loss of vitality.

*Dr. Smith*—Said he uses crowns largely, and sets them with gutta-percha. As the question of bridge-work naturally comes into this discussion, he wanted to say that he is entirely opposed to the practice which too commonly prevails, of sacrificing good natural crowns to make a foundation for setting bridge-work. He exhibited a case of this work done several years ago, where the occlusion of the jaws had been changed by building up the entire palatal surfaces of the superior incisors and cuspids with gold, and then the piece of bridge-work made, and it had done good service for several years.

*Dr. Baker*—Uses crowns, and often combines with them what is known as the Bing method, but in these cases he always sets the bar with amalgam to prevent subsequent rocking.

Drs. Smith, Hawes, and Baker, were appointed to consider the question of change of date for the annual meeting.

The Society then adjourned.

---

* See article on Gutta-percha, by Dr. T. T. Moore, in the number for July, 1885, p. 449.

